# Validation of Reference Genes via qRT-PCR in Multiple Conditions in Brandt’s Voles, *Lasiopodomys brandtii*

**DOI:** 10.3390/ani11030897

**Published:** 2021-03-21

**Authors:** Lin Tian, Yan Chen, Da-Wei Wang, Xiao-Hui Liu

**Affiliations:** 1 State Key Laboratory for Biology of Plant Diseases and Insect Pests and Key Laboratory of Weed and Rodent Biology and Management, Institute of Plant Protection, Chinese Academy of Agricultural Sciences, Beijing 100193, China; tianlin@caas.cn (L.T.); dwwang@ippcaas.cn (D.-W.W.); 2 State Key Laboratory of Integrated Management of Pest Insects and Rodents, Institute of Zoology, Chinese Academy of Sciences, Beijing 100101, China; Yanc1130@163.com

**Keywords:** reference gene, tissues, developmental stages, photoperiod

## Abstract

**Simple Summary:**

This study validated the stability of the expression profiles of nine common candidate reference genes (*Gapdh*, *Hprt1*, *β-actin*, *PPIA*, *Rpl13a*, *Tbp*, *Sdha*, *Hmbs,* and *B2M*) using qRT-PCR in different tissues, developmental stages, and photoperiods. None of these genes were suitable as optimal reference genes at 4 weeks postnatal in different tissues. Under different developmental stages in the hypothalamus, *B2M* for males and *Rpl13a* for females were suitable as reference genes. Under different photoperiods in the hypothalamus, none of the selected genes were suitable as reference genes at 6 weeks postnatal, *β-actin* and *PPIA* were the optimal reference genes at 12 weeks postnatal, while *Hprt1*, *β-actin*, *PPIA*, *Hmbs*, and *B2M* were excellent reference genes at 24 weeks postnatal.

**Abstract:**

The choice of optimal reference gene is challenging owing to the varied expression of reference genes in different organs, development stages, and experimental treatments. Brandt’s vole (*Lasiopodomys brandtii*) is an ideal animal to explore the regulatory mechanism of seasonal breeding, and many studies on this vole involve gene expression analysis using quantitative real-time polymerase chain reaction (qRT-PCR). In this study, we used the method of the coefficient of variation and the NormFinder algorithm to evaluate the performance of nine commonly used reference genes *Gapdh*, *Hprt1*, *β-actin*, *PPIA*, *Rpl13a*, *Tbp*, *Sdha*, *Hmbs,* and *B2M* using qRT-PCR in eight different tissues, five developmental stages, and three different photoperiods. We found that all nine genes were not uniformly expressed among different tissues. *B2M* and *Rpl13a* were the optimal reference genes for different postnatal development stages in the hypothalamus for males and females, respectively. Under different photoperiods in the hypothalamus, none of the selected genes were suitable as reference genes at 6 weeks postnatal; *β-actin* and *PPIA* were the optimal reference genes at 12 weeks postnatal; *Hprt1*, *β-actin*, *PPIA*, *Hmbs*, and *B2M* were excellent reference genes at 24 weeks postnatal. The present study provides a useful basis for selecting the appropriate reference gene in *Lasiopodomys brandtii*.

## 1. Introduction

The variation in gene expression level is a direct biomarker that can be used to capture individual responses to a changing environment. Quantitative real-time polymerase chain reaction (qRT-PCR) is a widely utilized method [1,2] with the advantage of easy-accessibility, fast processing, high accuracy, and high sensitivity in detecting the gene expression level [3]. In qRT-PCR, at least one reference gene is used as an internal control gene for the normalization of gene expression using 2^−ΔΔCt^ method [4], which can minimize the variations in RNA concentration and quantity, the amplification reaction, and a variety of treatments.

An ideal reference gene is considered to be expressed at a constant level under all different conditions; such genes are often referred to as housekeeping genes. However, even housekeeping genes are differentially expressed across various tissues, developmental stages, and treatments [5]. Barber (2005) reported that a 15-fold difference in *Gaphd* mRNA copy number was observed between the highest- and lowest-expressing human tissues [6]. *β-actin* and *Gapdh* displayed a significantly variable expression in bronchoalveolar lavage fluid cells and endobronchial biopsy tissue between controls and patients [7,8]. The expression of *β-actin* by qRT-PCR showed a dose-dependent inhibition in matrigel treatment [9]. Therefore, a common tactic for selecting an optimal reference gene should be aimed at particular experimental conditions [10].

Currently, *β-actin*, *Gapdh*, *PPIA*, *Rpl13a*, *Tbp*, *Sdha*, *B2M*, and *Hprt1* are commonly used as reference genes for qRT-PCR in humans [11], animals [12,13], and plants [3]. In seasonal breeding rodents, the photoperiod is considered as the most predictable indicator to mediate seasonal reproduction. The hypothalamus plays a vital role in photoperiodic response in seasonal breeders. Type 2 and type 3 iodothyronine deiodinase (Dio2 and Dio3) in the hypothalamus balance the local thyroid hormone levels to regulate the seasonal shifts of gonadal activity. Brandt’s vole (Lasiopodomys brandtii) is an ideal animal to explore the regulatory mechanism of seasonal breeding, and many studies on this vole involve gene expression analyses using qRT-PCR. *β-actin*, *Gapdh*, and *Hprt1* have been used for gene expression studies of seasonal breeding [14] and energy homeostasis [15] via qRT-PCR in Brandt’s vole. However, suitable reference genes in different tissues, in the hypothalamus under various development stages, and in the hypothalamus under different photoperiods have not been identified for the normalization of the target gene stage’s expression levels by qRT-PCR.

In the present study, we evaluated the stability of nine candidate reference genes in different tissues, developmental stages, and other photoperiod conditions. To further validate our results, type 2 iodothyronine deiodinase expression profiles were analyzed under different photoperiod conditions.

## 2. Materials and Methods

### 2.1. Sample Collection

Brandt’s voles were collected from a laboratory colony maintained at the Institute of Plant Protection, Chinese Academy of Agricultural Sciences (CAAS). Food and filtered tap water were provided ad libitum; the cotton-nesting material was available in the cage. Ambient temperature and relative humidity were continuously held at 23 ± 2 °C and 50 ± 10%, respectively. Sampled tissues were immediately frozen and stored at −80 °C until RNA extraction. The sample information is listed in Table 1.

### 2.2. RNA Extraction and cDNA Synthesis

RNA was extracted via the Direct-zol RNA MiniPrep (ZYMO RESEARCH, Irvine, CA, USA) kit. The integrity and concentration of RNA were assessed by 1% agarose gel electrophoresis and a nanodrop2000 spectrophotometer (Thermo Fisher Scientific, Waltham, MA, USA), respectively. The exact quantity of RNA (466 μg for different tissues, 840 μg for various developmental stages, and 450 μg for other photoperiod conditions) was reverse transcribed to the first-strand cDNA using the Fast Quant RT Kit (TransGen Biotech, Beijing, China).

### 2.3. Selection of Candidate Reference Genes

According to previous studies in rodents, nine candidate reference genes were selected [16,17,18,19]. *Gapdh* and *β-actin* are classic reference genes in various species, and *Hprt1*, *Rpl13a*, *Tbp*, *Sdha*, *Hmbs*, *PPIA*, and *B2M* are high-frequency reference genes in rodents. The cDNAs of these genes were cloned according to the respective cDNAs of Microtus ochrogaster and used for further primer design. GeneBank Accession Numbers were attained, including MT913769 (*Gapdh*), MT913770 (*Hprt1*), MT913772 (*β-actin*), MT913771 (*PPIA*), MT913773 (*Rpl13a*), MT913774 (*Tbp*), MT913775 (*Sdha*), MT913776 (*Hmbs*), and MT913777 (*B2M*).

### 2.4. qRT–PCR

Primers for qRT-PCR were designed by the Oligo 7.0 (OLIGO team, Colorado Springs, CO, USA) software and synthesized by Sangon Biotech Company (Beijing, China). After the quality test of primers according to the standard curve in qRT-PCR using SYBR Green PCR Master Mix (Thermo Fisher Scientific, Waltham, MA, USA), we used the BioMark™ HD System (Fluidigm Sciences Inc., South San Francisco, CA, USA) following Wang et al.’s method to assess mRNA levels of these candidate reference genes [14]. We used 48.48 Dynamic Array IFC (Fluidigm Sciences Inc., South San Francisco, CA, USA), which can load 48 samples and 48 assays. To exclude possible system errors, we performed a test of the same sample set using the same chip.

### 2.5. Statistical Analysis

The Shapiro–Wilk test was used for the normality test. The threshold cycle (Cq) value distribution of a candidate reference gene should be theoretically normal. Variations in the Cq value of each gene among different tissues, developmental stages, or photoperiods in individual assays were tested by one-way ANOVA or the Kruskal–Wallis test using SPSS version 19.0 (IBM Corp., Armonk, NY, USA) according to the normality test. *p* < 0.05 was considered statistically significant for all of the tests. The coefficient of variation (CV) was used to show the extent of the variability of candidates in all treatments in one sample set, with genes with lower CVs considered to be more stable.

The NormFinder algorithm (Aarhus University Hospital, Aarhus, Denmark) provides a stability value that estimates the most stable genes based on the estimation of intra- and intergroup variations. Intragroup variation means variation among individuals of the same treatment, and intergroup variation means variation between treatments. NormFinder compares the expression stability of genes across tissues, developmental stages, and photoperiods. This software focuses on finding reference genes with lower intra- and inter-group variations. We used the intergroup variation of 0.5 recommended by NormFinder as the stability threshold [20]. The gene with the lowest stability value is considered to show a stably expressed pattern and vice versa.

The stability of gene expression was analyzed by both CV and the NormFinder algorithm [20]. The result of the Normfinder algorithm was obtained by R software version 3.6.1 (R core Team, Vienna, Austria) with codes (https://www.moma.dk/normfinder-software. accessed on 14 June 2020).

## 3. Results

### 3.1. Specificity and Amplification Efficiency of Primers

The single lane with the expected size on an agarose gel and the single peak in the melting curve of qRT-PCR, as shown in Figures S1 and S2, indicated the high specificity of each primer pair. The PCR efficiency ranged from 95.443 to 107.581%, the melting temperatures were all 62 °C, and the R-squared value ranged from 0.983 to 1 (Table 2), indicating that these primers satisfied the standard requirements of qRT-PCR.

### 3.2. Expression Features in Different Tissues

We analyzed the intergroup variations and Coefficients of Variation (CV) of candidate genes and combined them with the NormFinder algorithm to screen out optimal reference genes under different treatments. After analyses of the intergroup Cq value variations, candidates with no significant difference in intergroup Cq values were further analyzed using the NormFinder algorithm. We also considered the results of the normality test, CV, and stability value provided by the NormFinder algorithm to determine the final suitable reference gene(s). In this assay, we analyzed the expression feature of candidate reference genes among different organs or tissues. Of nine candidates, seven genes, including *Gapdh*, *β-actin*, *PPIA*, *Rpl13a*, *Sdha*, *Hmbs*, and *B2M*, were not detected, with a significant intergroup difference (*p* > 0.05; Table 3), while the genes *PPIA*, *Hmbs*, and *B2M* displayed a significantly skewed distribution (*p* < 0.05; Table 3). The gene *Sdha* showed the lowest CV and the highest rank in the NormFinder algorithm (Table 4). These results indicated that *Sdha* was the best of the candidates when tissues were collected at postnatal day (PND) 4 w under the natural photoperiod condition. However, compared to the expected intergroup variation value (0.5) for an optimal reference gene using the NormFinder algorithm, the value of 1.95 indicated that *Sdha* could not be considered a good reference gene.

### 3.3. Expression Features at Different Developmental Stages

We tested the expression levels of nine candidates in male hypothalamus tissues collected at different developmental stages. Of the nine candidates, only *B2M* did not exhibit a significant intergroup difference (*p* > 0.05; Table 5). This indicates that most candidates were variably expressed at different developmental stages in the male hypothalamus and were not suitable for use as reference genes. *B2M* displayed a normal distribution and a low value of CV and could be selected as a reference gene under these circumstances. Here, we did not test its performance using NormFinder, as it requires at least three genes to perform the test.

In female samples, we detected three of nine candidates, including *PPIA*, *Rpl13a*, and *B2M*, that displayed no significant intergroup difference (*p* > 0.05; Table 6). Of the three genes, *B2M* displayed the lowest CV and the worst intergroup variation by the NormFinder algorithm, and *Rpl13a* displayed a similar CV and the highest rank by the NormFinder algorithm (Table 6 and Table 7). This indicates that *Rpl13a* is an optimal reference gene in females in different developmental stages.

### 3.4. Expression Features under Different Photoperiod Conditions

We analyzed the expression features of candidate genes in hypothalamus tissues at the developmental stage of PND 6w, which experienced different photoperiod conditions. Of nine candidates, only *Tbp* displayed no significant intergroup difference (*p* > 0.05; Table 8). These results indicate that most candidate genes are sensitive to photoperiod conditions at the developmental stage of PND 6w. The Shapiro–Wilk test indicated a significantly skewed distribution of *Tbp*. This result indicates that even *Tbp* is not a good choice as a reference gene under different photoperiod conditions, although it displays a relatively small intergroup variation.

At the developmental stage of PND 12w, all candidate genes displayed no significant intergroup difference under different photoperiod conditions and no significantly skewed distribution (Table 9). This indicates that these genes are not sensitive to photoperiod conditions when Brandt’s voles develop to PND 12w. *PPIA* and *β-actin* displayed a lower intergroup variation than the threshold of 0.5 using the Normfinder algorithm (Table 10). This indicates that these two genes can be selected as the optimal reference genes under different photoperiod conditions.

At the developmental stage of PND 24w, all candidate genes displayed no significant intergroup difference under different photoperiod conditions and no significantly skewed distribution (Table 11). By the NormFinder algorithm, five of nine candidates, including *Hprt1*, *β-actin*, *PPIA*, *Hmbs*, and *B2M*, displayed a lower intergroup variation than the threshold of 0.5 (Table 12). These genes could be selected as the reference genes in future studies. Compared to the developmental stage of PND 12w, more candidate genes were expressed stably under different photoperiod conditions when Brandt’s voles developed to PND 24w.

### 3.5. Validation of the Selected Reference Genes under Different Photoperiod Conditions

**Figure 1:**
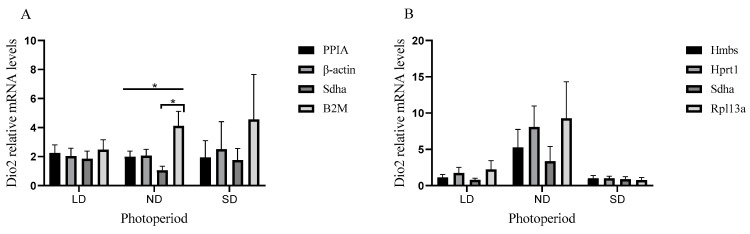
Identification of the stability of reference gene expression. Expression levels of a target gene, *Dio2*, in hypothalamus tissues at the developmental stage of PND 12w (A) and 24w (B) were normalized by using the top two most stable and least stable candidates under individual developmental stages. Bars represent the means and SEM. *p* < 0.05, *.

To validate the stability of the candidate reference genes, the mRNA relative expression levels of a target gene *Dio2* were normalized by the top two most stable and least stable candidates in their individual developmental stages. Under different photoperiods, no optimal reference genes were selected at PND 6. At PND 12 (Figure 1A), there was no significant difference between *Dio2* expression normalization by the top two most stable reference genes, *PPIA* and *β-actin,* in long-day photoperiod (LD), natural-day photoperiod (ND), or short-day photoperiod (SD) condition. One-way ANOVA indicated a significant difference between *PPIA*, *β-actin*, *Sdha*, and *B2M* under the ND condition (*p* = 0.015), and the result normalized using *Sdha* was significantly lower than *B2M* under ND condition (independent t-test, *p* = 0.018). Although no significant difference was found under the SD condition, the fold ratio of the mean of the expression level of the result between using *PPIA* and *B2M* was up to 2.35. Similarly, at PND 24w (Figure 1B), there was no significant difference between the top two most stable reference genes, *Hmbs* and *Hprt1*, in LD, ND, or SD condition. No significant difference was found analyzed by one-way ANOVA, whereas the fold ratio of the mean of the expression level of the result between using *Hmbs* and *Rpl13a* was up to 1.76. These results indicated the qPCR results normalized by the top two most stable reference genes were beyond those normalized by the top two least stable reference genes under different photoperiod conditions.

## 4. Discussion

In this present study, we evaluated the expression levels of nine genes that are usually selected as the reference genes in qRT-PCR. We found that all the tested genes displayed a higher intergroup difference in various tissues at PND 4w. *B2M* and *Rpl13a* were the optimal reference genes for different postnatal development stages in the hypothalamus of males and females, respectively. Under different photoperiods in the hypothalamus, none of the selected genes were suitable as reference genes at PND 6w; *β-actin* and *PPIA* were the optimal reference genes in the hypothalamus after PND 12w, more optimal reference genes (*Hprt1*, *β-actin*, *PPIA*, *Hmbs*, and *B2M*) were found at PND 24w.

We analyzed the data using the coefficient of variation (CV) and NormFinder, which assessed the candidate reference genes’ stability using different algorithms. By using CV and intergroup statistical analysis, the candidates with a normal distribution, no significant intergroup variation, and a lower CV were found to be relatively stable, which can be further analyzed using NormFinder. The NormFinder algorithm combines intragroup and intergroup variations to calculate the candidate reference genes’ stability value for validation. The reference gene with the lowest intergroup variation and stability value is the most stable. Only the candidates who pass the evaluation using these two algorithms can be selected as an optimal reference gene.

None of these genes were suitable as optimal reference genes in different tissues at PND 4w. This may be due to the fact that most dynamic changes in gene expression occur before puberty, during which different tissues have different gene expression profiles and types [21,22,23]. For example, *Tbp* was highly expressed in the testis compared with other organs at PND 4w in our study because it is involved in the transcription of the androgen receptor [24]. Androgen receptors are highly expressed in the testis during puberty [25], and PND 4w is around puberty in Brandt’s voles, which reach sex maturation after 4–6 weeks [26]. These results remind us that other candidate reference genes besides the selected candidates in this study need to be selected for validation.

We next investigated the stability of the nine selected genes in the hypothalamus under different developmental stages and photoperiod conditions. In this study, *B2M* in males and *Rpl13a* in females were not developmentally dynamic genes and had housekeeping functions in the postnatal development stages. Although *B2M* and *Rpl13a* are not classic reference genes, they are also listed as suitable reference genes in other studies. For instance, *B2M* is the most stable reference gene in the developmental stage of the half-smooth tongue sole (*Cynoglossus semilaevis*) [27], and *Rpl13a* is the most stable reference gene in the developmental stage of Diaphania caesalis (*Lepidoptera Pyralidae*) [28]. Under different photoperiods, the expression of all nine genes was affected by photoperiods for males going through puberty, while these candidates’ stability increased along with the development. It was supported by the increased number of suitable reference genes from PND 12w to PND 24w. We verified these results using a photoperiod-sensitive gene, *Dio2*. We found that different reference genes could lead to different statistical results. This implies the necessity of the careful selection of reference genes.

We found that all the tested common reference genes were unsuitable for normalizing the qRT-PCR assay between different tissues in Brandt’s voles going through puberty. In the postnatal development of the hypothalamus, *B2M* and *Rpl13a* were suitable reference genes for males and females, respectively. Several tested genes, including *Hprt1*, *β-actin*, *PPIA*, *Hmbs*, and *B2M*, could be used as optimal reference genes to normalize the adult stage’s photoperiod treatment in the hypothalamus of Brandt’s voles.

## 5. Conclusions

Overall, our study shed light on the selection of an optimal reference gene in Brandt’s vole (*Lasiopodomys brandtii*) in multiple conditions. None of the nine tested genes can be selected as reference genes across all present treatment conditions; more candidate reference genes should be added for further analysis. Under different developmental stages in the hypothalamus, *B2M* for males and *Rpl13a* for females were not developmentally dynamic genes. Under different photoperiod conditions in the hypothalamus, none of the selected genes were suitable as reference genes at PND 6w. *β-actin* and *PPIA* were the optimal reference genes at PND 12w, and *Hprt1*, *β-actin*, *PPIA*, *Hmbs*, and *B2M* were excellent reference genes at PND 24w. The present study also reminds us of the difficulty of finding real reference genes, because all genes vary their expression at some stages. We need to carefully select reference genes according to the particular experimental conditions involved.

## Figures and Tables

**Table 1:** Sample information.

Sample Sets	Tissue Type	Postnatal Day	Sex	Photoperiod	Samples of Different Treatments	Total
Tissues	Hypothalamus, pituitary, heart, kidney, adrenal gland, small intestine, bladder, and testes	4 week(w)	male	Voles were raised under the natural photoperiod conditions in Beijing city. All samples were dissected in May.	3,3,3,3,3,3,3,3	24
Developmental stages	Hypothalamus	2 w, 4 w, 8 w, 9 months (m), 20 m	female	Voles were raised under the natural photoperiod conditions in Beijing city. Males and females were dissected between May and June.	8,8,7,8,8	39
	Hypothalamus	2 w, 4 w, 8 w, 9 m, 20 m	male		7,8,8,8,8	39
Photoperiod conditions	Hypothalamus	6 w	male	Voles were raised under different photoperiod conditions, including Light: Dark = 16:8; Light: Dark = 8:16; and the natural photoperiod conditions from September to next March in Beijing city. The age of the newborns was equivalent to the period of treatment.	4,3,6	13
	Hypothalamus	12 w	male		6,5,7	18
	Hypothalamus	24 w	male		4,5,4	13

**Table 2:** Test of primer quality.

Gene Name	Primers (5′-3′)	Amplicon Size(bp)	PCR Efficiency	R-Squared Value
*Gapdh*	GCTGCCCAGAACATCATCCCTG	126	96.685	0.999
	GACGACGGACACATTGGGGGTA			
*Hprt1*	TGACACTGGTAAAACAATGCAGACT	110	95.576	0.999
	ACCCAACACTTCGAGAGGTCC			
*β-actin*	GCTCTCTTCCAGCCTTCCTTCCTG	112	96.471	0.999
	GTGTTGGCGTACAGGTCCTTGCGG			
*PPIA*	TGGTGGGTAAGAAGCCCGCAA	110	95.443	1
	GGAAGCCATGGAGCGTTTTGGA			
*Rpl13a*	CATGCTGCCCCACAAGACCA	150	97.145	0.987
	GCAAACTTCCTTGTAGGCTTCAG			
*Tbp*	CCCTATGACCCCGATCACTCC	165	107.581	0.983
	GCAGCAAACCGCTTGGGATTAT			
*Sdha*	AATTACAAGGGGCAGGTGCTGAA	139	100.345	0.991
	TACGAGGTCCAATAGGGAATTTGC			
*Hmbs*	TGCCAGAGAAAAGTGCGGTG	102	100.429	0.993
	TGAGGTTGCCCCGAATACTCC			
*B2M*	GTTACACACACCACTCTGAAGGAAC	115	98.056	0.997
	TTAAACTGGTCCAAATGAAGCATCT			
*Dio2*	[14]			

**Table 3:** Expression features in different tissues at postnatal day (PND) 4W under the natural photoperiod condition analyzed by SPSS.

Gene Name	*p*-Value by Shapiro-Wilk Test	*p*-Value of Intergroup Variation	Mean Cq	Standard Deviation (SD) Cq	CV	Rank
*Gapdh*	0.37	0.230	15.85	2.22	13.99	6
*Hprt1*	0.24	0.039	19.76	2.54	12.86	5
*β-actin*	0.16	0.055	15.64	2.23	14.26	7
*PPIA*	0.01	0.138	13.58	1.96	14.46	8
*Rpl13a*	0.15	0.194	15.19	2.28	15.02	9
*Tbp*	0.30	0.011	22.29	2.60	11.67	3
*Sdha*	0.09	0.125	19.06	1.85	9.73	1
*Hmbs*	0.01	0.080	21.48	2.26	10.52	2
*B2M*	0.01	0.051	18.50	2.21	11.93	4

**Table 4:** Expression features in different tissues at PND 4W under the natural photoperiod condition analyzed by Normfinder.

Gene Name	Intergroup Variation	Intragroup Variation	Stability Value	Rank
*Gapdh*	3.15	0.54	0.89	4
*Hprt1*				
*β-actin*	2.09	0.68	0.95	5
*PPIA*	2.62	0.36	0.64	3
*Rpl13a*	2.01	0.76	0.98	6
*Tbp*				
*Sdha*	1.95	0.42	0.73	1
*Hmbs*	2.80	0.59	0.75	2
*B2M*	5.67	1.46	1.53	7

**Table 5:** Expression features in male hypothalamus tissues at different developmental stages analyzed by SPSS.

Gene Name	*p*-Value by Shapiro-Wilk Test	*p*-Value of Intergroup Variation	Mean Cq	SD Cq	CV	CV Rank
*Gapdh*	7.95 × 10^−2^	5.20 × 10^−5^	12.16	0.97	7.99	8
*Hprt1*	5.00 × 10^−1^	2.75 × 10^−2^	14.63	0.50	3.43	1
*β-actin*	1.19 × 10^−1^	2.44 × 10^−6^	13.30	1.09	8.19	9
*PPIA*	6.07 × 10^−1^	6.61 × 10^−4^	10.91	0.56	5.11	6
*Rpl13a*	1.86 × 10^−1^	6.15 × 10^−3^	12.55	0.57	4.53	3
*Tbp*	1.99 × 10^−2^	1.47 × 10^−4^	19.27	0.89	4.59	4
*Sdha*	1.11 × 10^−1^	6.90 × 10^−5^	15.76	0.96	6.09	7
*Hmbs*	1.61 × 10^−1^	1.79 × 10^−4^	18.33	0.92	5	5
*B2M*	8.20 × 10^−1^	6.30 × 10^−1^	18.20	0.73	4.01	2

**Table 6:** Expression features in female hypothalamus tissues at different developmental stages analyzed by SPSS.

Gene Name	*p*-Value by Shapiro-Wilk Test	*p*-Value of Intergroup Variation	Mean Cq	SD Cq	CV	CV Rank
*Gapdh*	2.74 × 10^−3^	6.44 × 10^−3^	12.08	1.18	9.75	9
*Hprt1*	3.16 × 10^−4^	1.56 × 10^−2^	14.54	0.83	5.70	2
*β-actin*	2.06 × 10^−3^	5.58 × 10^−4^	13.15	1.26	9.60	8
*PPIA*	5.38 × 10^−4^	5.36 × 10^−2^	10.85	0.84	7.74	7
*Rpl13a*	1.42 × 10^−4^	6.68 × 10^−2^	12.53	0.84	6.72	4
*Tbp*	1.93 × 10^−5^	1.35 × 10^−3^	19.17	1.18	6.13	3
*Sdha*	1.45 × 10^−3^	7.70 × 10^−3^	15.63	1.17	7.50	6
*Hmbs*	2.50 × 10^−4^	4.18 × 10^−3^	18.30	1.27	6.91	5
*B2M*	5.63 × 10^−1^	1.47 × 10^−1^	18.11	1.03	5.68	1

**Table 7:** Expression features in female hypothalamus tissues at different developmental stages analyzed by NormFinder.

Gene Name	Intergroup Variation	Intragroup Variation	Stability Value	Rank
*Gapdh*				
*Hprt1*				
*β-actin*				
*PPIA*	0.78	0.26	0.25	2
*Rpl13a*	0.23	0.15	0.13	1
*Tbp*				
*Sdha*				
*Hmbs*				
*B2M*	1.01	0.66	0.4	3

**Table 8:** Expression features in hypothalamus tissues at the developmental stage of PND 6w under different photoperiod conditions analyzed by SPSS.

Gene Name	*p*-Value by Shapiro-Wilk Test	*p*-Value of Intergroup Variation	Mean Cq	SD Cq	CV	CV Rank
*Gapdh*	0.25	8.97 × 10^−4^	17.31	2.18	12.61	8
*Hprt1*	0.04	3.95 × 10^−2^	19.82	1.85	9.33	5
*β-actin*	0.22	1.29 × 10^−2^	16.80	1.53	9.08	4
*PPIA*	0.42	2.08 × 10^−2^	14.06	1.32	9.41	6
*Rpl13a*	0.46	6.42 × 10^−4^	20.12	2.47	12.26	7
*Tbp*	0.01	5.87 × 10^−2^	22.08	1.98	8.97	3
*Sdha*	0.26	4.55 × 10^−4^	22.58	2.94	13.02	9
*Hmbs*	0.96	4.07 × 10^−2^	23.73	1.93	8.15	2
*B2M*	0.31	3.60 × 10^−2^	19.31	1.53	7.94	1

**Table 9:** Expression features in hypothalamus tissues at the developmental stage of PND 12w under different photoperiod conditions analyzed by SPSS.

Gene Name	*p*-Value by Shapiro-Wilk Test	*p*-Value of Intergroup Variation	Mean Cq	SD Cq	CV	CV Rank
*Gapdh*	0.94	0.59	17.91	1.78	9.96	9
*Hprt1*	0.61	0.19	20.12	1.29	6.43	4
*β-actin*	0.79	0.53	17.26	1.10	6.35	3
*PPIA*	0.22	0.58	14.14	1.15	8.17	6
*Rpl13a*	0.84	0.75	20.89	1.89	9.07	7
*Tbp*	0.67	0.31	22.13	1.31	5.94	1
*Sdha*	0.62	0.41	23.30	2.15	9.23	8
*Hmbs*	0.58	0.25	24.21	1.58	6.53	5
*B2M*	0.10	0.22	19.42	1.19	6.12	2

**Table 10:** Expression features in hypothalamus tissues at the developmental stage of PND 12w under different photoperiod conditions analyzed by NormFinder.

Gene Name	Intergroup Variation	Intragroup Variation	Stability Value	Rank
*Gapdh*	0.68	0.67	0.47	4
*Hprt1*	1.21	0.83	0.6	6
*β-actin*	0.29	0.57	0.36	2
*PPIA*	0.2	0.61	0.35	1
*Rpl13a*	0.8	1.11	0.6	7
*Tbp*	0.63	0.7	0.45	3
*Sdha*	1.35	1.12	0.73	9
*Hmbs*	1.2	0.35	0.52	5
*B2M*	1.4	0.56	0.61	8

**Table 11:** Expression features in hypothalamus tissues at the developmental stage of PND 24w under different photoperiod conditions analyzed by SPSS.

Gene Name	*p*-Value by Shapiro-Wilk Test	*p*-Value of Intergroup Variation	Mean Cq	SD Cq	CV	CV Rank
*Gapdh*	0.88	0.74	18.54	1.97	10.61	9
*Hprt1*	0.85	0.95	19.82	1.09	5.52	1
*β-actin*	0.34	0.70	16.97	1.26	7.40	6
*PPIA*	0.34	0.80	13.99	0.98	7.02	5
*Rpl13a*	0.95	0.88	21.56	1.70	7.87	7
*Tbp*	0.29	0.65	21.78	1.41	6.47	3
*Sdha*	0.35	0.76	23.71	2.21	9.31	8
*Hmbs*	0.20	0.82	24.07	1.62	6.73	4
*B2M*	0.98	0.91	19.00	1.08	5.70	2

**Table 12:** Expression features in hypothalamus tissues at the developmental stage of PND 24w under different photoperiod conditions analyzed by NormFinder.

Gene Name	Intergroup Variation	Intragroup Variation	Stability Value	Rank
*Gapdh*	0.72	0.64	0.35	7
*Hprt1*	0.49	0.45	0.25	2
*β-actin*	0.41	0.39	0.26	3
*PPIA*	0.09	0.56	0.27	4
*Rpl13a*	1.22	0.92	0.47	8
*Tbp*	0.62	0.58	0.33	6
*Sdha*	1.08	1	0.5	9
*Hmbs*	0.35	0.26	0.21	1
*B2M*	0.44	0.55	0.27	5

## Supplementary Materials

The following are available online at https://www.mdpi.com/2076-2615/11/3/897/s1, Figure S1: 1.5 % agarose gel electrophoresis is exhibiting Amplification products of 9 candidate reference genes from *L. brandtii* by normal PCR, Figurs S2: Specificity detection of primers for each candidate genes by melting curves.

## References

[1] Kubista M., Andrade J.M., Bengtsson M., Forootan A., Jonak J., Lind K., Sindelka R., Sjoback R., Sjogreen B., Strombom L. (2006). The real-time polymerase chain reaction. Mol. Asp. Med..

[2] Vandesompele J., De Preter K., Pattyn F., Poppe B., Van Roy N., De Paepe A., Speleman F. (2002). Accurate normalization of real-time quantitative RT-PCR data by geometric averaging of multiple internal control genes. Genome Biol..

[3] Wang H., Wen H., Li Y., Zhang K., Liu Y. (2018). Evaluation of potential reference genes for quantitative RT-PCR analysis in spotted sea bass (Lateolabrax maculatus) under normal and salinity stress conditions. PeerJ.

[4] Schefe J.H., Lehmann K.E., Buschmann I.R., Unger T., Funke-Kaiser H. (2006). Quantitative real-time RT-PCR data analysis: Current concepts and the novel "gene expression’s CT difference" formula. J. Mol. Med..

[5] Nailis H., Coenye T., Van Nieuwerburgh F., Deforce D., Nelis H.J. (2006). Development and evaluation of different normalization strategies for gene expression studies in Candida albicans biofilms by real-time PCR. BMC Mol. Biol..

[6] Barber R.D., Harmer D.W., Coleman R.A., Clark B.J. (2005). GAPDH as a housekeeping gene: Analysis of GAPDH mRNA expression in a panel of 72 human tissues. Physiol. Genom..

[7] Glare E.M., Divjak M., Bailey M.J., Walters E.H. (2002). beta-Actin and GAPDH housekeeping gene expression in asthmatic airways is variable and not suitable for normalising mRNA levels. Thorax.

[8] Chapman J.R., Waldenstrom J. (2015). With Reference to Reference Genes: A Systematic Review of Endogenous Controls in Gene Expression Studies. PLoS ONE.

[9] Selvey S., Thompson E.W., Matthaei K., Lea R.A., Irving M.G., Griffiths L.R. (2001). Beta-actin--an unsuitable internal control for RT-PCR. Mol. Cell. Probes.

[10] Nolan T., Hands R.E., Bustin S.A. (2006). Quantification of mRNA using real-time RT-PCR. Nat. Protoc..

[11] Xiao J., Li X., Liu J., Fan X., Lei H., Li C. (2017). Identification of reference genes in blood before and after entering the plateau for SYBR green RT-qPCR studies. PeerJ.

[12] Niu G., Yang Y., Zhang Y., Hua C., Wang Z., Tang Z., Li K. (2016). Identifying suitable reference genes for gene expression analysis in developing skeletal muscle in pigs. PeerJ.

[13] Pérez R., Tupac-Yupanqui I., Dunner S. (2008). Evaluation of suitable reference genes for gene expression studies in bovine muscular tissue. BMC Mol. Biol..

[14] Wang D., Li N., Tian L., Ren F., Li Z., Chen Y., Liu L., Hu X., Zhang X., Song Y. (2019). Dynamic expressions of hypothalamic genes regulate seasonal breeding in a natural rodent population. Mol. Ecol..

[15] Zhang X.Y., Zhang Q., Wang D.H. (2011). Litter size variation in hypothalamic gene expression determines adult metabolic phenotype in Brandt’s voles (Lasiopodomys brandtii). PLoS ONE.

[16] Sakai H., Sato K., Kai Y., Shoji T., Hasegawa S., Nishizaki M., Sagara A., Yamashita A., Narita M. (2014). Distribution of aquaporin genes and selection of individual reference genes for quantitative real-time RT-PCR analysis in multiple tissues of the mouse. Can. J. Physiol. Pharm..

[17] Rueda-Martínez C., Fernández M.C., Soto-Navarrete M.T., Jiménez-Navarro M., Fernández B. (2016). Identification of Reference Genes for Quantitative Real Time PCR Assays in Aortic Tissue of Syrian Hamsters with Bicuspid Aortic Valve. PLoS ONE.

[18] Xu D., Liu A., Wang X., Zhang M., Zhang Z., Tan Z., Qiu M. (2018). Identifying suitable reference genes for developing and injured mouse CNS tissues. Dev. Neurobiol..

[19] Svingen T., Letting H., Hadrup N., Hass U., Vinggaard A.M. (2015). Selection of reference genes for quantitative RT-PCR (RT-qPCR) analysis of rat tissues under physiological and toxicological conditions. PeerJ.

[20] Andersen C.L., Jensen J.L., Orntoft T.F. (2004). Normalization of real-time quantitative reverse transcription-PCR data: A model-based variance estimation approach to identify genes suited for normalization, applied to bladder and colon cancer data sets. Cancer Res..

[21] Hou H., Uusküla-Reimand L., Makarem M., Corre C., Saleh S., Metcalf A., Goldenberg A., Palmert M.R., Wilson M.D. (2017). Gene expression profiling of puberty-associated genes reveals abundant tissue and sex-specific changes across postnatal development. Hum. Mol. Genet..

[22] Cardoso-Moreira M., Halbert J., Valloton D., Velten B., Chen C., Shao Y., Liechti A., Ascencao K., Rummel C., Ovchinnikova S. (2019). Gene expression across mammalian organ development. Nature.

[23] Palmert M.R., Boepple P.A. (2001). Variation in the timing of puberty: Clinical spectrum and genetic investigation. J. Clin. Endocrinol. Metab..

[24] Lavery D.N., McEwan I.J. (2006). The human androgen receptor AF1 transactivation domain: Interactions with transcription factor IIF and molten-globule-like structural characteristics. Biochem. Soc. Trans..

[25] Liu T.D., Yu B.Y., Luo F.H., Zhang X.L., Wu S., Liu L.H., Wu Y.J. (2012). Gene Expression Profiling of Rat Testis Development During the Early Post-Natal Stages. Reprod. Domest. Anim..

[26] Chen Y., Wang D., Li N., Hu X., Ren F., Hao W., Song Y., Liu X. (2019). Kinship analysis reveals reproductive success skewed toward overwintered Brandt’s voles in semi-natural enclosures. Integr. Zool..

[27] Liu C., Xin N., Yi Z., Jiang L., Zhai J., Zhang Q., Jie Q., Susanne K.E. (2014). Reference Gene Selection for Quantitative Real-Time RT-PCR Normalization in the Half-Smooth Tongue Sole (Cynoglossus semilaevis) at Different Developmental Stages, in Various Tissue Types and on Exposure to Chemicals. PLoS ONE.

[28] Wang Z., Meng Q., Zhu X., Sun S., Liu A., Gao S., Gou Y. (2020). Identification and Evaluation of Reference Genes for Normalization of Gene Expression in Developmental Stages, Sexes, and Tissues of Diaphania caesalis (Lepidoptera, Pyralidae). J. Insect Sci..

