# Supplementary material for: Validation of Reference Genes via qRT-PCR in Multiple Conditions in Brandt’s Voles, Lasiopodomys brandtii
Source: Animals (Basel). 2021 Mar 21;11(3):897. doi: 10.3390/ani11030897 (PMC8004067; doi:10.3390/ani11030897)
Supplement: Supplementary file 1 [file animals-11-00897-s001.zip › agarose_gel_electrophoresis_supplement_Figure_S1.docx]

**Figure S1.** 1.5 % agarose gel electrophoresis is exhibiting Amplification products of 9 candidate reference genes from *L. brandtii* by normal PCR
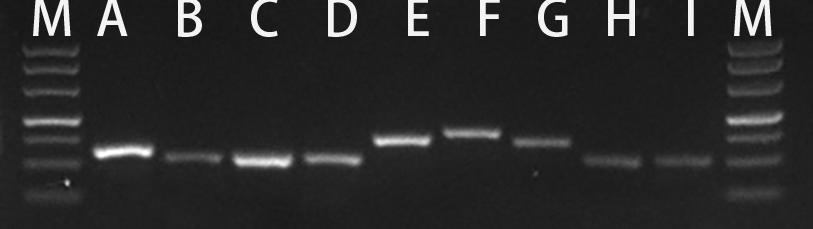


M: DL 500 maker. Lanes A, B, C, D, E, F, G, H and I were the genes *Gapdh*, *Hprt1,* *PPIA*, *β-actin*, *Rpl13a*, *Tbp*, *Sdha*, *Hmbs* and *B2M* from *L. brandtii*, respectively.
