# Supplementary material for: Validation of Reference Genes via qRT-PCR in Multiple Conditions in Brandt’s Voles, Lasiopodomys brandtii
Source: Animals (Basel). 2021 Mar 21;11(3):897. doi: 10.3390/ani11030897 (PMC8004067; doi:10.3390/ani11030897)
Supplement: Supplementary file 1 [file animals-11-00897-s001.zip › Melting_curves_supplement_Figure_S2.docx]

**Figure S2.** Specificity detection of primers for each candidate genes by melting curves


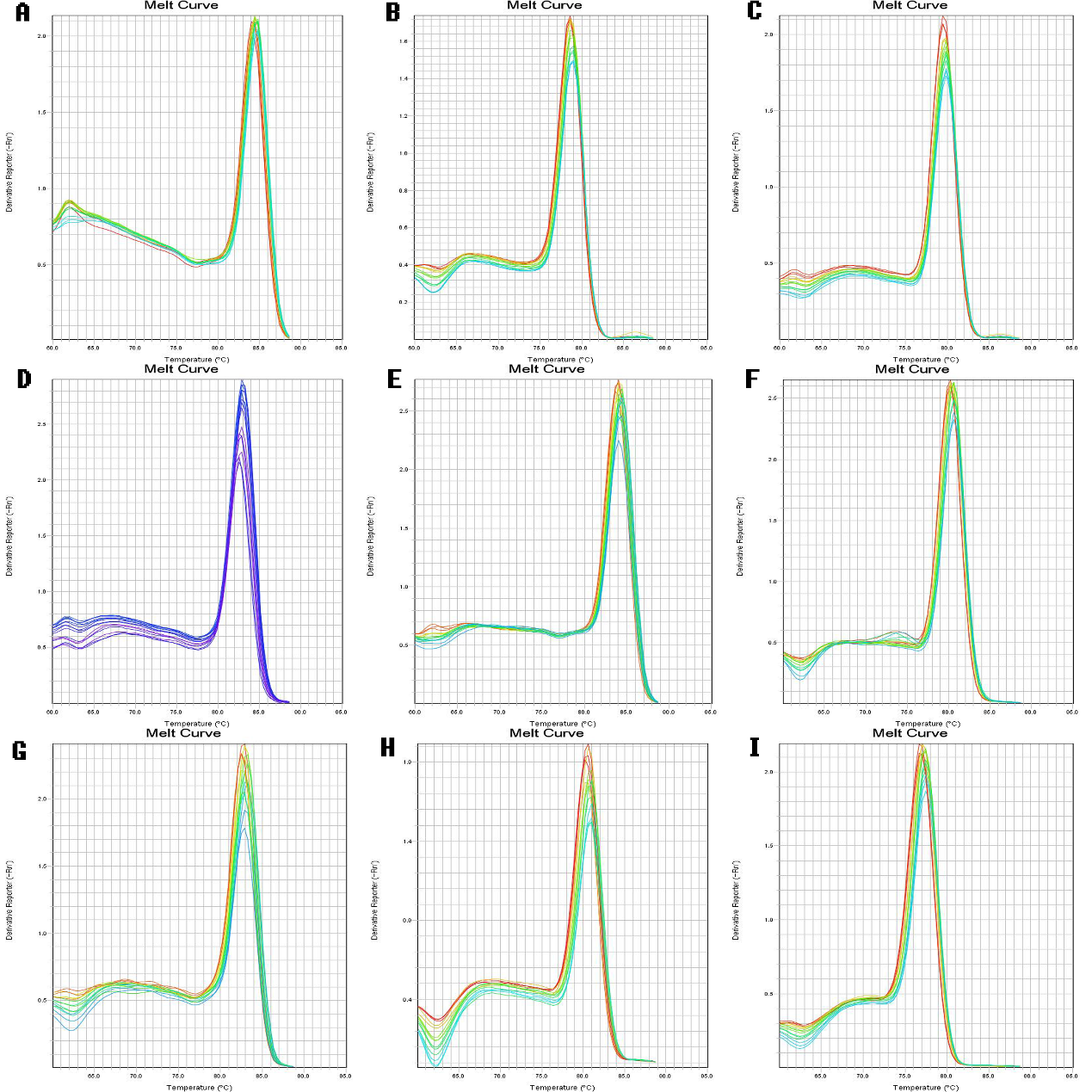


Dissociation curves with single peaks were generated from all amplicons for 9 candidate reference genes *Gapdh* (A); *Hprt1* (B); *PPIA* (C); *β-actin* (D); *Rpl13a* (E); *Tbp* (F); *Sdha* (G); *Hmbs* (H); *B2M* (I)
